# Evaluation of the Olli C + D biochemical analyser after over a year of use in enzymology

**DOI:** 10.1155/S1463924681000588

**Published:** 1981

**Authors:** G. Baraton, D. Grafmeyer, A. Capuano, L. Richard, J. Sofia

**Affiliations:** Laboratoire automatisdé Biochimie Clinique (Professeur J. Bertrand) Hopital Edouard Herriot Place d'Arsonval Lyon Cedex 2 69374 France


with carry-over or drift. Automation of these RIA assays has halved the man-hours normally required and eliminated the many errors which can occur in the manual analysis and clerical reporting of large batches of specimens.

One of the drawbacks of the system is that complete shutdown occurs when there is a printer failure. This fail-safe system is not essential when the analyser is connected to a laboratory computer. Another feature that could be improved is the sampler. A 40 specimen sampler is too small when large batches are being proceesed. Fewer plate changes would be required if the sampler size was increased to hold 60 or preferably 100 samples.

### Evaluation of the Olli C + D biochemical analyser after over a year of use in enzymology

**G. Baraton, D. Grafmeyer, A. Capuano, L. Richard and J. Sofia.**
*Laboratoire automatisé de Biochimie Clinique, (Professeur J. Bertrand), Hopital Edouard Herriot, Place d'Arsonval, 69374 Lyon Cedex 2, France*

To meet today's clinical requirements, clinical biochemistry laboratories must be increasingly automated. The required criteria for an automatic analyser are reliability; the ability to change chemical reactions easily and quickly; rapid determinations and low analysis cost.

It appears that the Olli C + D parallel analyser (Kone Oy instrument division Espoo, Finland) answers these criteria. A report on the Olli C system has already been published by Puuka and Pukka [1]. This evaluation, using the C + D system, deals with its intensive and daily use in enzymology over a period lasting more than one year.

### Description

The instrument is a parallel, multichannel, photometric analyser. The different phases of determination are performed in batches of 24 samples including blanks, standards and controls. The rate of analysis is 700 tests per hour with end point determinations of 360 kinetic measurements per hour – reaction time is 2½ minutes.

The analyser comprises three independent units, the sample diluter/processor, photometer (each controlled by microprocessor) and incubator. The sample change is performed by hand using disposable cuvettes in a thermostated block.

The Olli D Diluter measures 350 x 1100 x 740 mm and weighs 70 kg. It is made up of a dispensing tray with 24 cuvette blocks which fit in the Olli C photometer, eight reagent vessels, one block with 24 serum vials, an eight tip dispensing head and a digital display and keyboard. The diluter can be programmed by hand or by tape cassette for 30 analyses; it is possible to add serum and up to eight reagents distributed in one or several runs. Each analysis programme follows the sequence: 1. Attain reaction temperature (25, 30, 37°C). 2. Measure reagent (5 to 1000 μl by steps of 1μl) and position. 3. Measure and add sample. 4. Mix (5 to 180 seconds).

The photometer measures 330 x 520 x 740 mm and weighs 48 kg. It comprises a computer, a thermal printer and a thermostated measuring chamber (25°, 30°, 37°C). The light source is a zenon lamp. A quartz optic fibre system allows the simultaneous measurement of 24 cuvettes. Filters have a bandwidth of 8 to 15 nm. The smallest readable volume is 500 μl for end point determinations, and 300 μl for kinetic determinations.

The photometer can be programmed by hand or by tape cassette for 15 analyses which may be made in end point mode, with or without blank measurement, or in kinetic mode, with or without reagent or serum blanks. Kinetics determinations are made using linear regression of 12 to 24 measurements in 1 to 10 minutes. The results are printed with the name and units of the programmed analysis. Error messages are printed; eg, if the chosen initial absorbance limits have been exceeded or if there is non-linearity of reaction. In addition, it is possible to have printed the twelve or 24 absorbance measurements used for the calculation of the activity of each determination.

The Olli incubator can handle four thermostated blocks. Each block contains one heating element and temperature regulation is maintained via a water circulating system. The incubation period is determined by a timer and each block incubation time is terminated with an audible alarm.

### Material and methods

The evaluation was carried out according to the rules suggested by the French Society for Clinical Biology (SFBC) [2, 3].

### Dilution and measurement

Accuracy, within-block and day-to-day, was tested with a solution of drawing ink (Mars 745 from Staedler Germany) prepared as follows: 1.7 ml of ink as supplied was dissolved by gentle stirring in 1000 ml of distilled water and filtered in a buchner funnel through silicone paper. The solution was preserved with Merseptyl and stored at +4°C. Dilutions were made by the Olli D processor into Olli arcylic cuvettes and absorbances were read on the Olli C photometer at 405 nm. Measurements were verified by manual dilution followed by a reading using a Miniken spectrophotometer (Coultronic).

### Control of temperature

The control of the temperature variations between 25 and 37°C was tested by measuring the absorbance of a paranitrophenol solution, (15 mg in 1000 ml of Tris HCL 0.2 M, pH 6.8) at 405nm, Naudin *et al* [4]. The absorbances obtained at the chosen temperature were compared with those obtained using four thermostated analysers; Miniken (Coultronic), Cobas BIO (Roche,) PA 800 (Vitratron), ACP 5040 (Eppendorf). On the Olli C, the time needed to reach the set temperature of 30°C or 37°C with an analysis volume of 550 μl

(contained in the acrylic cuvette in the thermostated block) was followed by noting the decrease in the absorbance of the paranitrophenol solution. The temperatures of cuvette contents were also monitored with a Metrix temperature probe (HA 1159).

### Optical performance

This was tested according to the procedure of Rand [5]. Linearity and precision were tested using the following kits: UV-Trol (Biomerieux France) and Holnicob (Biotrol France).

### Method

All analyses were performed at either 30°C or 37°C. The instrument settings were those recommended by the manufacturer. Glutamic oxaloacetic transaminase (SGOT) and pyruvic transaminase (SGPT) were measured by the Wroblewski method at 37°C or by the recommended method of the SFBC at 30°C with the Biomerieux kits. Alkaline phosphatase (ALP) was measured following the recommended procedure of the German Society of Clinical Biochemstry (DGKC) at 37°C. Accuracy, within day and day to day, and carryover were tested using low, medium and high concentration control sera spiked where necessary with animal enzymes. These control sera were furnished by a local association operating in biological measurement quality control. (Pro Bio. Qual, Lyon, France).

### Results and discussion

### Accuracy and precision of the Olli D sample processor

The results are presented in Table 1. Precision expressed as the coefficient of variation for 24 determinations was never greater than 0.925% for the most important dilution (10 µl of drawing ink in 510 µl of water).

The measured absorbances varied directly with the amount of drawing ink added and were indistinguishable from those obtained by manual dilution. Day-to-day precision was 0.609% for the 1/12 dilution used routinely for SGOT and SGPT and 0.925% for the 1/30 dilution used routinely for ALP.

The absorbance of drawing ink is temperature independant. The maximum variation for a temperature range of 20 – 40°C and an absorbance of 1.690 was ± 0.002.

### Temperature control

With the different automatic analysers the solution of paranitrophenol gave an average inverse ratio of 0.029 absorbance units for an increase of 1°C. With the Olli C, the absorbances found were 1.948 ± 0.002 at 25°C and 1.595 ± 0.004 at 37°C, or 0.029 absorbance units/degree.

The desired termperature was reached rather slowly. If the blocks and cuvettes were preheated to the designated temperature of 37°C, the usual volume of para-nitrophenol (at the initial temperature of + 4°C) reached 37°C in 8 minutes in the Olli C and 7 minutes in the incubator (Figure 1). Similar results (not shown) were found for a set temperature of 30°C, the steady state was reached in 6 minutes for the Olli C, and 5 minutes for the incubator.

The block has to be manipulated outside the apparatus, and it is therefore important to know the rate of cooling. At a set temperature of 37°C, the block outside the apparatus cooled by 0.5°C during the first minute and then 1°C/minute during the following 5 minutes. Measurements were made with a temperature probe at ambient temperature.

To avoid this cooling when the blocks were manipulated outside the incubator for either the Olli C or the Olli D, the set temperature in the incubator was set slightly higher, at 30.2° for 30°C and 37.4° for 37°C.

### Accuracy and precision of the photometer

Linear absorption was maintained up to 2.2 units of absorbance at 340 nm and up to 2.8 at 405 nm. Accuracy and precision, tested at 340 nm with the UV Trol kit (stabilised solution of NADH) were both good. Similar results at 660 nm were found with the Holnicob-kit (cobalt nitrate).

### Analytical results precision

Within-day and day-to-day precision measurements, precision for SGOT measurements at 37°C are presented in Tables 2 and 3. The CV did not exceed 2.1% at any of the levels of serum activity studied. The SGPT and ALP values, measured at 37°C, (valued not shown) were similar. SFBC recommendations specify within-day precision for SGOT to be within 2.0% at the low level, 0.9% at the medium level and 1.9% at the high level of activity.

No carry over was detected when one row of eight high level control sera was followed by two rows of eight low level control sera.

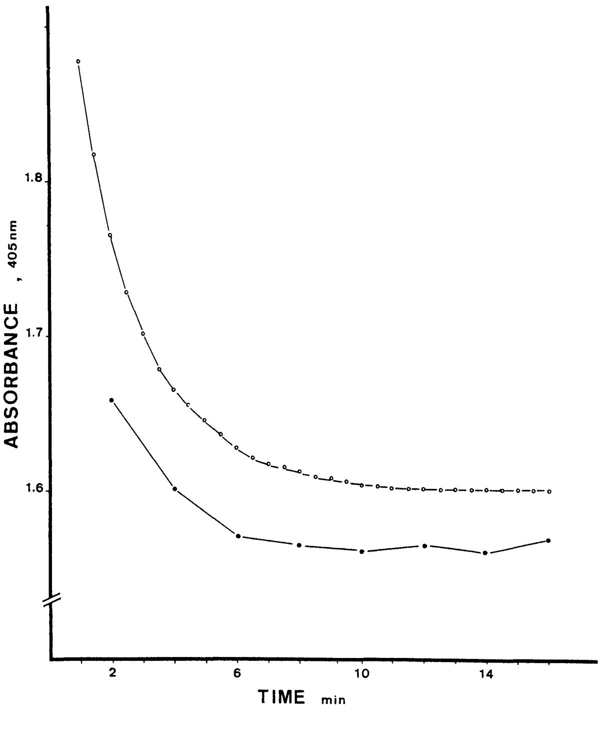

*Figure 1. The time to reach the ordered temperature of 37°C is followed by the decrease of absorbance of paranitrophenol at 405nm (see method section). Blocks and cuvettes were preheated to 37°C. The initial temperature of the paranitrophenol was 4°C. (o - Olli C; ● - incubator).*

**Table 1. Accuracy and precision of diluter Olli D. Results obtained using dilutions of drawing ink as described in Method Section.**

| Dilutions | 10/510 | 20/520 | 60/560 | 100/600 | 120/620 | 160/640 |
|---|---|---|---|---|---|---|
| Average absorbance (24 determinations) | 0.186 | 0.356 | 1.002 | 1.561 | 1.813 | 2.274 |
| CV% | 0.925 | 0.438 | 0.354 | 0.299 | 0.284 | 0.256 |

**Table 2. Within day precision for SGOT measurements Results obtained using protocol A established by the SFBC. [2] [3]**

| Controls | Number of determinations | Averages U/L | Standard deviation | CV % |
|---|---|---|---|---|
| Low level | 16 | 34.4 | 0.5 | 1.5 |
| Medium level | 16 | 57.1 | 0.8 | 1.4 |
| High level | 16 | 199.2 | 2.7 | 1.3 |

**Table 3. Between days precision for SGOT measurements Results obtained using protocol A established by the SFBC [2] [3].**

| Controls | Number of determinations | Average U/L | Standard deviation | CV % |
|---|---|---|---|---|
| Low level | 160 | 33.7 | 0.7 | 2.0 |
| Medium level | 160 | 56.5 | 1.1 | 1.9 |
| High level | 160 | 197.6 | 3.0 | 1.5 |

*Day-to-day precision and accuracy*

Sera with three levels of SGPT activity were assayed each month for one year. The results are shown in Figure 2 where each point represents two to five daily determinations. Similar results were found (not shown) for SGOT and ALP where the CV was never more than 7% at any considered serum level.

*Reliability*

No injury to operators was caused or seemed likely during the twelve month period of the test.

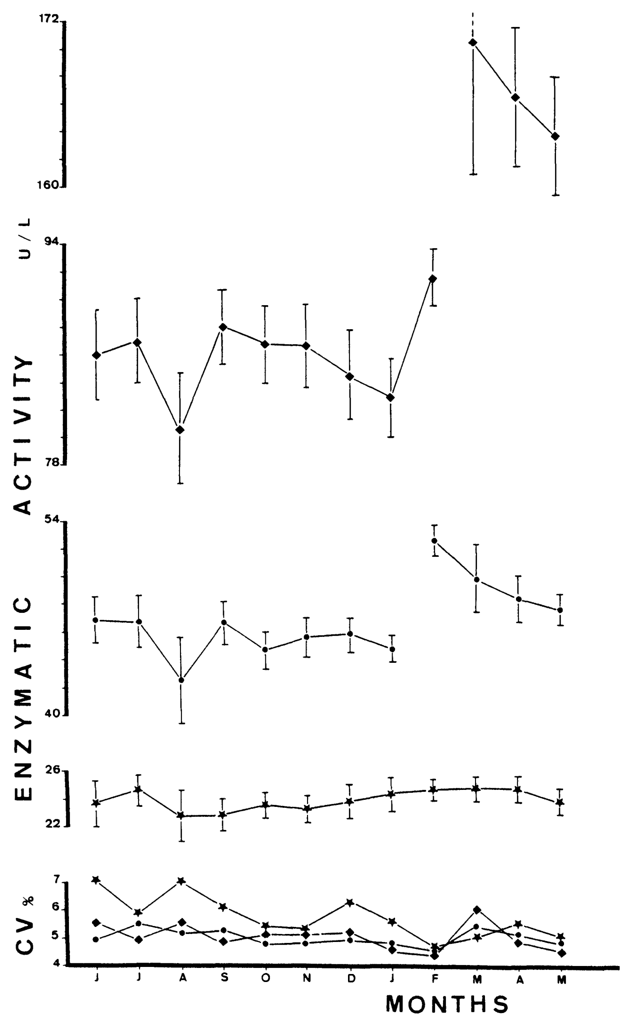

*Figure 2. Each point represents the average of two to five daily control determinations on sera with three levels of activity during a month. (* – low level; ● – medium level; ■ – high level).*

The electronics performed well but were very sensitive to even a short power interruption, so the analyser should be electrically stabilised to prevent the Olli D or Olli C of the computer memory being erased.

Systematic cleaning was carried out between each analysis and no problems were experienced with the needle obstruction.

Manual use of the keyboard and programming of the microprocessor Olli D and C was easy.

### Conclusion

The Olli C + D analyser with its incubator proved to be reliable and sufficiently precise. Its advantages are speed, end point or kinetics measurements for enzymology or immunoenzymology, and the absence of immobilisation of the Olli C or D during long incubation periods.

Each analytical step is displayed and many malfunctions and errors are indicated. Reliable results were obtained over a period of more than one year with 300 – 600 daily enzymatic determinations. A block for reactions volumes of 300 $\mu$l is being developed by Kone and should lower reagent costs.

ACKNOWLEDGEMENTS

The authors wish to thank Mrs Bonnet Paulien for reading the manuscript and Mr Panigoni (Kone Oy Instrument) for his help during this work.

REFERENCES

[1] Puuka, R. and Puuka, M. (1980), *J Automat Chem* **2**, 153-158
[2] Collombel, C. (1977), *Ana Biol Clin*, **35**, 167-192
[3] Grafmeyer,D. (1978) Etude des performances globales d'apres le protocole A S.F.B.C. in Societe Francaise de Biologie Clinique. Mesure des Activites enzymatiques en Biologie Clinique. Grafmeyer, G., Collombel, C., Dingeon, B., Fournier, M., Mathieu, N., Varennes, J.P., Eds Association Amicale des Etudiants en Pharmacie, Lyon, FRANCE, pp 129-137
[4] Naudin, C. and Bailly, M. (1978), Etude de la thermostatation des analyseurs d'enzymes in Societe Francaise de Biologie Clinique. Grafmeyer, D., Collombel, C., Dingeon, B., Fournier, M., Mathieu, M., Varennes, J.P., Eds Association Amicale des Etudiants en Pharmacie, Lyon, FRANCE, pp 145-147
[5] Rand, R.N. (1969), *Clin Chem*, **15**, 839-863

